# Eyesight: a study of the staff of a dental school

**DOI:** 10.1038/bdjopen.2017.8

**Published:** 2017-05-19

**Authors:** Nicholas P Chandler, Andrew R Gray, Colleen M Murray

**Affiliations:** 1 Department of Oral Rehabilitation, Sir John Walsh Research Institute, Faculty of Dentistry, University of Otago, Dunedin, New Zealand; 2 Department of Preventive and Social Medicine, Dunedin School of Medicine, University of Otago, Dunedin, New Zealand

## Abstract

**Objectives/Aims:**

The aim was to investigate the vision of all 90 dentally qualified staff at a dental school.

**Materials and Methods:**

Ethical approval was obtained and a questionnaire-based survey conducted. Data were screened and analysed using Stata 13.1. The *χ*^2^ and Fisher’s Exact tests were used to test for significance with an alpha level of 0.05.

**Results:**

The participation rate was 95.6%. Most of the teachers (92%) considered their eyesight was satisfactory to practice dentistry. Of the 97% who had been tested at some stage, 15% had their eye examination due to sight deterioration with 22% needing correction. Almost two-thirds were myopic and a third were hyperopic. Forty-nine per cent wore spectacles only, with about a quarter of this group alternating between spectacles and contact lenses. Of those with corrected vision, 80% followed their optometrist’s recall advice. Four participants reported that they were colour blind. While 4% had had laser-eye surgery, a further 27% were interested in this. Magnification was used by 72% with no significant differences between genders, age of staff member, place of qualification or registration status. Most of the staff (81%) thought that screening of dental student’s eyesight should be mandatory, and regular eye examinations as a condition of dental practice was supported by 67%.

**Discussion:**

The number of teachers reporting recent vision tests was encouraging; nevertheless, a worrying 8% surveyed were unsure if their eyesight was satisfactory for work. The commonest vision problem was myopia, with almost half of the teachers wearing spectacles. It is clear that visual standards for dentistry would be helpful. Magnification use was high, with many non-users indicating their intention to buy loupes.

**Conclusions:**

Within the limits of this study the teachers were conscientious regarding their eye care, irrespective of their training and age. There was strong support for the mandatory testing of vision for all dentists and especially dental students.

## Introduction

Dentistry is visually demanding. Irreversible procedures are carried out in the mouth and excellent eyesight is necessary for these and other tasks such as clinical examination and interpreting radiographs. A dental teachers performance could be influenced by visual defects and so problems should be identified early, with regular eye examinations strongly encouraged. A recent survey revealed that over 31% of dental students were unsure if their eyesight was satisfactory to practice dentistry.^1^ Very little scientific research has been carried out on the visual acuity of dentists and the influence of different optical devices and magnification on their performance.^2^ The aim of this study was to investigate dentally qualified teachers to learn more about their vision and eye care habits.

## Materials and methods

Ethical approval was obtained from the University of Otago Human Ethics Committee (reference D14/304). The study included all 90 dentally qualified teachers at the School of Dentistry, Dunedin, New Zealand (NZ) in 2014. The group included dentists registered in the General Dental Practice (GDP) scope or as specialists by the Dental Council (NZ) together with some unregistered (mainly overseas trained) dentists. The latter were restricted to simulation teaching. A participant information sheet described the study and indicated that completing a questionnaire would imply consent. Completed forms were returned to a designated area to maintain anonymity. Those who chose to participate were entered in a prize draw.

An 18-question survey was developed using a multiple-choice format with space for additional information if the applicable option was not listed or if the staff member wished to add further information. It was pretested for clarity and lack of ambiguity. The first section covered demographic details, while the remainder assessed self-reported eyesight status, factors that had influenced them to attend for an eye examination, their perceptions of recommended timing of recall appointments and compliance with their optometrists’ recommendations. It also investigated vision aids such as loupes and attached lights.

Stata 13.1 (StataCorp LP, College Station, TX, USA) was used for all analyses and two-sided *P*<0.05 was considered statistically significant. Mann–Whitney *U* and Kruskal–Wallis tests were used as appropriate.

## Results

Responses were received from 86 (95.6%) of the staff (Table 1). Seventy-seven (90%) were registered and 9 (10%) unregistered. Of the 77 registered dentists, 36 (42%) were specialists and 19 (22%) postgraduate students. Fifty-nine per cent were male and 41% female. Some 92% thought their eyesight was satisfactory for dentistry, but 8% were unsure. The mean time since qualification was 20.4 years (range 2–45 years), corresponding to the age of the participants (mean 44.2 years, range 26–68 years).

Three teachers reported never having had an eye test. They were a male NZ graduate aged 32 undertaking postgraduate studies, a 31-year-old female postgraduate aged 31 who studied dentistry in Fiji and a 53-year-old male Sri Lankan graduate working in the GDP scope. Amongst the 83 teachers (97%) who had ever had an eye examination, 10 of them (15%) said they were motivated by having noticed a change in eyesight (Table 2). Sixty-two per cent had undergone an eyesight examination in the past year, and among corrective lens users 72% reported that their optometrist recommended check ups annually or every 2 years. Some 23% reported that they had either not had a recommendation or could not remember the advice given.

Short sightedness (myopia) was noted by 62% with long sightedness (hyperopia) much less common (33%). Astigmatism was reported by 31%. Four respondents (5%) reported that they believed their vision to be colour defective while three (4%) were unsure.

Corrective lens use by the participants was a feature of 49%, with the mean duration of lens wearing almost 20 years. No participants reported sole use of contact lenses, but alternating spectacle and contact lens wear was common (22%).

Of the 85 responses to questions on use of magnification, 61 (72%) used loupes, with 95% considering this improved visualisation and optimised clinical results (Table 3). With respect to magnification, there were no significant differences between genders (*P*=0.471), age of teacher (*P*=0.672), place of qualification (*P*=0.524) or registration status (*P*=0.632). Magnification did, however, differ significantly by teacher status (GDP, specialist or postgraduate student, *P*=0.006), with higher levels of use amongst general dentists (90%) compared to specialists (53%) (*P*=0.001) and with postgraduate students in between (78%) and not statistically significantly different from either other group (both *P*⩾0.138). There was no evidence that the strength of magnification used by loupe users differed by gender (*P*=0.060), age (*P*=0.173), place of qualification (*P*=0.686), registration status (*P*=0.453) or by teacher status (*P*=0.113). Almost one half used loupes with 2.5× or 3.0× magnification and exactly one half of the staff had loupes with an attached light. Expense was a consideration for 19% of the non-loupe users. An improvement in posture with loupes was cited by 49%.

There was strong support for mandatory eye examinations as a registration requirement (67%) with no significant differences by gender, country of qualification, age, status or registration held by the educators. There was even greater support (81%) for mandatory eyesight tests for dental students at the start of their programme, with no significant difference between teacher status. The importance of vision being satisfactory for work and the time since the most recent eye examination also did not differ significantly between types of teacher.

## Discussion

While a potential weakness of this study may be the self-reported nature of the data, research confirms an association between measured visual function and self-reported visual ability.^3^ The response rate was a very pleasing 95.6%, and it was reassuring that only three of the staff had never had their eyes tested. Nevertheless, seven (8%) were not sure if their vision was satisfactory for dental work.

The prevalence of myopia in the general population varies greatly, with significant ethnic differences.^4^ In our teacher group, 62% reported that they were myopic. The association between level of education and prevalence of myopia has been well documented^5,6^ with prevalence increasing from 64.1% in those with primary education to 85.0% in those with tertiary education.^7^ In Norway, the prevalence of myopia in medical students (63.9%) is almost twice as high as in the general population (35.0%).^8^

The influence of near-work on the development and progression of myopia has been investigated^9^ and the prevalence of myopia in the general population of Nordic countries is 30%.^10^ Time spent on practical near-work activities, reading as well as working at computer displays were investigated by these authors, with all except computer use influencing the development of myopia.

Adult onset myopia is really a form of hyperopia, best described as presbyopia. This age change is a concern in dentistry; it has been suggested that practitioners’ eyes should be examined every 2 years from age 40 years onwards.^11^ The natural visual acuity varies greatly between individuals,^2^ and to some extent acuity problems and deterioration in near vision may be overcome by using magnification.^2,11^ The prevalence of self-reported hyperopia in our group of dental educators was 33% and of astigmatism 31%.

The three teachers in our survey (3.5%) who reported never having had an eye examination had very varied backgrounds. The anonymity of the study precludes further investigation; however, in a 2007 survey of 247 UK dentists, 18 (7%) had not been examined.^11^ It is possible that our teaching group was more concerned about the quality of their vision; nevertheless, it is concerning that one of the untested respondents in our survey was over 50 years old, an age at which some suggest eye testing should be carried out at less than 2-year intervals.

Shade matching is a prerequisite for successful aesthetic results. Red–green deficiencies affect colour matching in the yellow region important to dentistry. Colour-defective persons cannot be expected to match the shades of teeth comparably with those of normal-vision.^12^ Routine colour-vision testing of dental students has been advocated and remains a current topic of debate.^13,14^ Colour-vision deficiencies affect mostly males with an incidence of approximately 8% for males and 0.5% for females. Four of our teachers were aware of being colour blind (all males) with a further two males and one female being unsure if they had this defect. The prevalence of colour deficiencies among dentists and dental technicians has been reported to range from 7.8 to 10.0%.^13^ Clearly it would be valuable for a dental teacher to know if he or she has this visual defect, as it may impact on teaching ability. For those with a known colour-vision problem, the help of an assistant or the use of a digital shade-taking device is possible.

Amongst the teaching staff who were spectacle and/or contact lens wearers, 27% were interested in laser surgery; this contrasts with the 69.3% of dental students from the same school who reported an interest in these procedures.^1^

In a study of dental school faculty at Temple University, Philadelphia, USA, 73% believed magnification should be a programme requirement.^15^ The most common magnification used by our staff was 2.5× (49%), the same magnification being popular in a UK study.^16^ Among a group of NZ dental students the use of loupes increased from 2% in the first clinical year to 48% in the final year, with 55% considering them too expensive.^1^ To a lesser degree cost was a significant factor in non-use by 19% of our educators, a finding similar to a UK study^16^ where cost was the most important factor governing their purchase. Nevertheless, 24% of our non-user educators did intend to purchase them. The advantages of loupes include improved accuracy of caries diagnosis,^17^ better evaluation of marginal discrepancies in restorations^18^ and benefits to posture.^11,19,20^

It was interesting to find a significant difference in use across the teacher groups. The high level of GDP teacher uptake (90%) might represent their value for patient treatment done part-time outside the school. The postgraduates also reported high use of loupes (78%). Many would have received operating microscope training and their loupe use could represent the magnification available on most undergraduate teaching clinics.

Exactly one half of our teachers used loupes with an attached light. The advent of lightweight batteries and very efficient LED lamps is increasing their popularity.

Dental treatments are carried out in a challenging, restricted and dark environment. Perhaps visual standards should be drawn up and eyesight testing (with correction if necessary) made a requirement? Most of our teaching staff (67%) thought that regular eyesight examinations should be mandatory as a condition of practice, but as 23% had not had a recall recommendation or could not remember receiving advice a regular reminder from the optometrist may be helpful. There are many professions, for instance aviation, where visual standards have been set. In dentistry patient safety cannot be ignored. As 8% of the group were unsure if their eyesight was satisfactory for work, and with this figure exceeding 31% among their students,^1^ this is a concern. Nevertheless, minor and correctable visual defects should not preclude individuals from a dental career.^21^

### Conclusion

The teachers were mostly very conscientious concerning their eye care and their uptake of magnification loupes was high. Screening of all dental professionals would help ensure that dental teachers, students and their patients could have confidence regarding their vision. Within the limitations of this study, there was strong support for mandatory eye testing for all dentists and especially for dental students.

## Figures and Tables

**Table 1:** Demographics, participating teaching staff (*N*=86)

*Variable*		n	*%*
Sex	Male	51	59
	Female	35	41
Country of primary qualification	NZOther	4937	5743
Status	General dentist	31	36
	Specialist	36	42
	Student	19	22
NZ registered	Yes	77	90
	No	9	10
			
		*Mean (s.d.)*	*Minimum/maximum*
Age	(Years)	44.2 (12.7)	26/68
Qualified	(Years since)	20.4 (12.5)	2/45

Abbreviation: NZ, New Zealand.

**Table 2:** Eye examinations and corrective lens use

*Variable*	n	*%*
*Ever had eyes tested*		
Yes	83	97
No	3	3
		
*Time since last test (among those who have been tested)*		
⩽1 year	51	62
2–3 years	20	24
4–5 years	4	5
>5 years	7	9
Missing	1	
		
*Reason for examination (multiple responses, among those who have been tested)*		
Change in eyesight	10	15
Part of a medical examination	4	6
Reminder from optometrist	7	10
Needed new spectacles	15	22
Felt it was overdue	11	16
Routine annual check-up	24	35
Getting headaches	1	1
Missing	15	
		
*Corrective lens use (among those who have been tested)*		
None	24	29
Spectacles only	40	49
Contact lenses only	0	0
Both	18	22
Missing	1	
		
*Optometrist recommendation for check-ups (among those who wear lenses)*		
Annual	22	39
Up to every 2 years	19	33
More than 2 years	3	5
No recommendation/can not remember	13	23
Missing	1	
		
*Follow optometrist’s advice (among lens wearers)*		
Yes	44	80
No	11	20
Missing	3	
		
*Eyesight status (among lens wearers)*		
Short-sighted (myopia)	34	62
Long-sighted (hyperopia)	18	33
Astigmatism	17	31
Presbyopia	2	4
Need progressive lenses	1	2
Don't know	2	4
Missing	3	
		
*Had laser-eye surgery*		
Yes	3	4
No	80	96
		
*Interested in laser surgery (among those who have not had and wear lenses)*		
Yes	15	27
No	41	73
Missing	2	
		
	*Mean (s.d.)*	*Minimum/maximum*
*Duration of lens wearing (among lens wearers)*		
(Years)	19.8 (12.9)	1/50
Missing	1	

**Table 3:** Dental teacher specific questions

*Variable*	n	*%*
*Frequency eyes should be examined*		
Annually or more often	36	42
2 yearly	36	42
3 yearly	2	2
4 yearly or less often	7	8
As needed	4	5
Missing	1	
		
*Own vision satisfactory for work*		
Yes	78	92
Not sure	7	8
Missing	1	
		
*Colour-vision defect*		
Yes	4	5
No	78	92
Unsure	3	4
Missing	1	
		
*Support mandatory regular exams for dentists*		
Yes	57	67
No	28	33
Missing	1	
		
*Support mandatory exam for students*		
Yes	69	81
No	16	19
Missing	1	
		
*Use loupes*		
Yes	61	72
No	24	28
Missing	1	
		
*Reasons for not using (multiple responses, among those not using loupes)*		
Do not need	17	81
Yet to purchase (planned)	5	24
Too expensive	4	19
Should be provided	1	5
Made nauseous	1	5
Missing	3	
		
*Reasons for using (multiple responses, among those using loupes)*		
Optimise clinical results/improve visualisation	58	95
Address eye accommodation	7	11
Improve posture	30	49
Better enjoyment	1	2
Course requirement	1	2
		
*Magnification (among those using loupes)*		
2×	4	7
2.5×	30	49
2.8×	1	2
3×	16	26
3.25×	1	2
3.5×	3	5
4×	5	8
4.5×	1	2
		
*Feature a light (among those using loupes)*		
Yes	30	50
No	30	50
Missing	1	
		
	*Median (IQR)*	*Minimum/Maximum*
Duration (among those using loupes)	5 (8)	0/30

Abbreviation: IQR, interquartile ratio.
